# Time trends in the incidence of essential tremor: Evidences from UK and France primary care data

**DOI:** 10.3389/fneur.2022.987618

**Published:** 2022-09-20

**Authors:** Ippazio Cosimo Antonazzo, Sara Conti, Davide Rozza, Carla Fornari, Caroline Eteve-Pitsaer, Claire Paris, Laurène Gantzer, Dennis Valentine, Lorenzo Giovanni Mantovani, Giampiero Mazzaglia

**Affiliations:** ^1^Research Centre on Public Health (CESP), University of Milano-Bicocca, Monza, Italy; ^2^Cegedim, Boulogne-Billancourt, France; ^3^Cegedim, London, United Kingdom

**Keywords:** essential tremor, THIN® database, epidemiology, United Kingdom, France

## Abstract

**Introduction:**

Although essential tremor (ET) is considered a common adult movement disorder, evidence on its incidence is still scant. This study aims at estimating ET incidence in two European countries, namely, the UK and France.

**Methods:**

Incident cases of ET were identified within the Health Improvement Network (THIN®) database between 1st January 2014 and 31 December 2019. Yearly crude and standardized incidence rates (IR) were estimated across the study period for both countries. Poisson regression models were built to assess temporal trends in IRs and differences between sexes and age classes.

**Results:**

In total, 4,970 and 4,905 incident cases of ET were identified in the UK and France, respectively. The yearly average crude IR (per 100,000 person-years) was 18.20 (95%CI: 15.09–21.32) in UK and 21.42 (17.83–25.00) in France, whereas standardized ones were 19.51 (18.97–20.01) and 19.50 (18.97-20.05). Regression analyses showed slightly increasing trends in both countries, higher incidence among males, and a significant increase with age. Yearly average IR increased from 3.96 (0.95–6.97) and 5.28 (1.12–9.44) in subjects aged <20 years to 49.27 (26.29–72.24) and 51.52 (30.19–72.86) in those aged >80 year in UK and France.

**Conclusions:**

Standardized ET incidence was comparable in the UK and France, showing a slight increase in both countries, reporting a higher value among people aged 60 years and older. This study outlines the need to conduct future studies to estimate the burden of ET in terms of disease control and healthcare resource utilization.

## Introduction

Essential tremor (ET) is the most common adult movement disorder observed in clinical practice and represents a frequent problem reported by individuals seeking neurologic consultation (1). Although ET is often regarded as a benign condition, patients may experience functional disability with a consequently diminished quality of life (2, 3). ET patients might also experience cognitive impairment, problems with balance, sleep dysregulation, anxiety, and depression (4).

For many decades, there was no widely accepted uniform ET definition. In addition, the potential overlap of ET and Parkinson's disease clinical features has challenged the ability to diagnose ET, contributing to diagnostic errors and inappropriate treatment (5, 6). Recently, the International Parkinson and Movement Disorder Society (MSD) proposed a new formal definition of ET based on two axes: the first focused on clinical features, whereas the second on etiology (2). MSD also defined ET as an isolated tremor syndrome, characterized by bilateral upper limbs action tremor, with a duration of at least 3 years, with or without a tremor in other locations, and in the absence of other neurologic signs such as parkinsonism, ataxia or dystonia (2, 7).

Great variability emerges from the scant epidemiological literature concerning the prevalence of ET: in 2020, a review estimated the global prevalence at 0.32% (95% CI: 0.12–0.91), ranging from 0.2% in Singapore to 8.6% in Spain (8). Similarly, recent studies reported significant geographical variability, with estimated prevalence ranging from 0.56% in North America to 5.42% in Africa, for a cohort with an average age of 57 years (8–10). Marked heterogeneity between reported ET estimates was largely due to variations in case definition and study design of the observed studies, generally using a questionnaire-based approach within a limited population sample.

So far, only two studies have estimated ET incidence, and among them only one was based in Europe, precisely in Spain, and it was focused only on individuals aged 65 years and older (11, 12). Therefore, considering the paucity of European data on ET incidence in the general population, it is crucial to produce new epidemiological evidence to plan future health interventions. This study aims to evaluate the incidence of ET in the UK and France, between 2013 and 2020, by using two large independent primary care databases extracted from The Health Improvement Network (THIN®) network.

## Methods

### Study design and data sources

This was a retrospective, descriptive study conducted by using primary healthcare data from the UK and France. The study was approved by the THIN® Scientific Research Committee (SRC) on 6th July 2021 (SRC reference 21-014).

The THIN® database (13), is a large standardized European database of fully anonymized electronic medical records collected from general practices that have joined the THIN® network. Information on symptoms, diagnoses, interventions, and referrals to secondary care are coded according to the Read code in the UK and the International Classification of Diseases, 10th revision (ICD10) in France. UK data were collected from about 400 general practices, representing around 6% of the UK population. Several published reports have demonstrated the representativeness of the collected information in terms of patients' demographics, the prevalence of chronic conditions, and mortality rates (13, 14). French data were collected from a pool of about 2,000 GPs and were representative of the French population in terms of age, gender, and geographic location (15). For each patient we had access to all diagnoses recorded by general practitioners (GPs), whether they were the main reason for a visit or the justification of a therapeutic-diagnostic intervention.

### Study population and case definition

All patients actively registered in general practice between 1st January 2014 and 31 December 2019, both in the UK and France were considered. The access to the general practitioner is regulated differently in the two countries: general practices in the UK, who are considered the gatekeeper, take charge of a patient and regulate all his accesses to health services (16), while in France patients can choose different GPs as required. Therefore, a subgroup of GPs was identified by the THIN® network as representative of the French population. In light of these considerations, we included the whole population recruited in the UK database and only those patients referred to representative GPs in France. In these two cohorts, all individuals reporting at least one of the following diagnosis codes during the study period were identified: Essential tremor (ICD10/France: G25.0); Essential and other specified forms of tremor (Read code/UK: F131.00); Benign essential tremor (Read code/UK: F131.00); Essential and other specified forms of tremor NOS (Read code/UK: F131z00). Individuals were included only if they had at least 3 years of database history prior to the date of the first coded ET diagnosis (Index date). This inclusion criterion, which follows the current ET definition requiring the symptoms to persist for at least 3 years before the diagnosis, limited the possibility of including prevalent cases, that were diagnosed before joining practices participating in THIN® (17, 18). Patients with dystonia, ataxia, Parkinson's disease, or parkinsonism diagnosed at any time before the index date were excluded from the selected cohorts (Supplementary Tables 1A,B). Selected subjects were identified as incident cases of ET and the index date was used as a proxy for the date of onset.

### Statistical analysis

Demographic characteristics of incident cases at ET onset were described in terms of frequency and percentage or mean and standard deviation as appropriate, and they were compared between France and the UK through Chi-square tests for categorical variables and *T*-tests for continuous ones.

Yearly-specific and yearly-average crude incidence rates were estimated across the whole study period for both the UK and France. Sex- and age-specific rates were computed by stratifying the population using the following age classes: <20 years, 20–39 years, 40–59 years, 60–79 years, ≥80 years. An exact 95% CI was computed assuming a Poisson distribution.

Person-years at risk for the denominators of incidence rates were approximated using the average active population of each year, computed as the average number of active registered patients from January 1st of the year of interest and December 31^st^ of the same year.

Direct standardization was applied for total and sex-specific rates, using the European population on 1 January 2020 as a reference, and 95% CI were computed assuming a Poisson distribution and using the normal approximation method (19).

To assess temporal trends in the incidence rates and highlight significant differences between sexes and among age classes, cases and the corresponding average population were stratified by calendar year, sex, and age class. Thereafter, for each country a Poisson regression model was built, accounting for overdispersion, with the following form:


$$\begin{array}{l} \log[E\left(Y_{ijk})\right]=\beta_{0}+\sum_{k=1}^{K-1}\beta_{r}I_{age_{k}}+\beta_{K}year+\;\beta_{K+1}I_{M}\\ \;+\log\left(pop_{ijk}\right) \end{array}$$

where *Y*_*ijk*_ represent the number of incident cases observed in the *i*th year, within the *j*th sex and the *k*th age-class, *I*_*ag*_*e*__*k*__ is an indicator variable for the *k*th age-class, *K* is the total number of age-classes, *year* is a continuous variable for the calendar year, *I*_*M*_ is an indicator variable for male sex, *pop*_*ijk*_ is the average population in the *i*th year, within the *j*th sex and the *k*th age-class. From these models, adjusted incidence rate ratios (IRR) for each independent variable, together with 95%CI, were estimated.

To ascertain temporal trends in the sex- or age-specific incidence rate, we carried out similar models but adding the interaction terms between sex and calendar year and age and calendar year.

All analyses were carried out with statistical software SAS version 9.4 (SAS Institute, Cary, NC, USA) and R version 4.0.5 (R Project for Statistical Computing, www.R-project.org). Confidence intervals were computed using the *exactci* package (20).

### Sensitivity analyses

We carried out two sensitivity analyses to establish the potential impact of diagnosis misclassification (i.e., patients with Parkinson's disease or parkinsonism previously misdiagnosed with ET) on the incidence estimates.

We re-evaluated ET incidence after excluding those patients who had a diagnosis of Parkinson's disease or parkinsonism within the first year or the first 3-years following the ET diagnosis, and those who did not have a 1- or 3-year follow-up period following such diagnosis, respectively. The second analysis was limited to the period 2014–2017, to guarantee the minimum follow-up period.

## Results

A total of 4,970 (27,303,661 estimated person-years of follow-up) and 4,905 (22,901,847 estimated person-years of follow-up) individuals were selected in the UK and France cohorts, respectively. The mean (SD) age at ET onset was 60.7 (19.4) vs. 61.0 (19.9) in the UK and France, respectively. The proportion of females was 49.2% in the UK and 53.5% in France (Table 1).

**Table 1:** Demographic characteristics of ET incident case, by country.

**Patients'**	**UK**	**France**	***P*-value**
**characteristics**	**(*N* = 4970)**	**(*N* = 4905)**	
**Age**			
Mean (SD)	60.7 (19.4)	61 (19.9)	0.177
**Age classes**			<0.001
<20	239 (4.8%)	222 (4.5%)	
20–39	595 (12%)	596 (12.2%)	
40–59	949 (19.1%)	1011 (20.6%)	
60–79	2519 (50.7%)	2278 (46.4%)	
≥80	668 (13.4%)	798 (16.3%)	
**Gender**			
Females	2443 (49.2%)	2624 (53.5%)	<0.001

**Figure 1:**
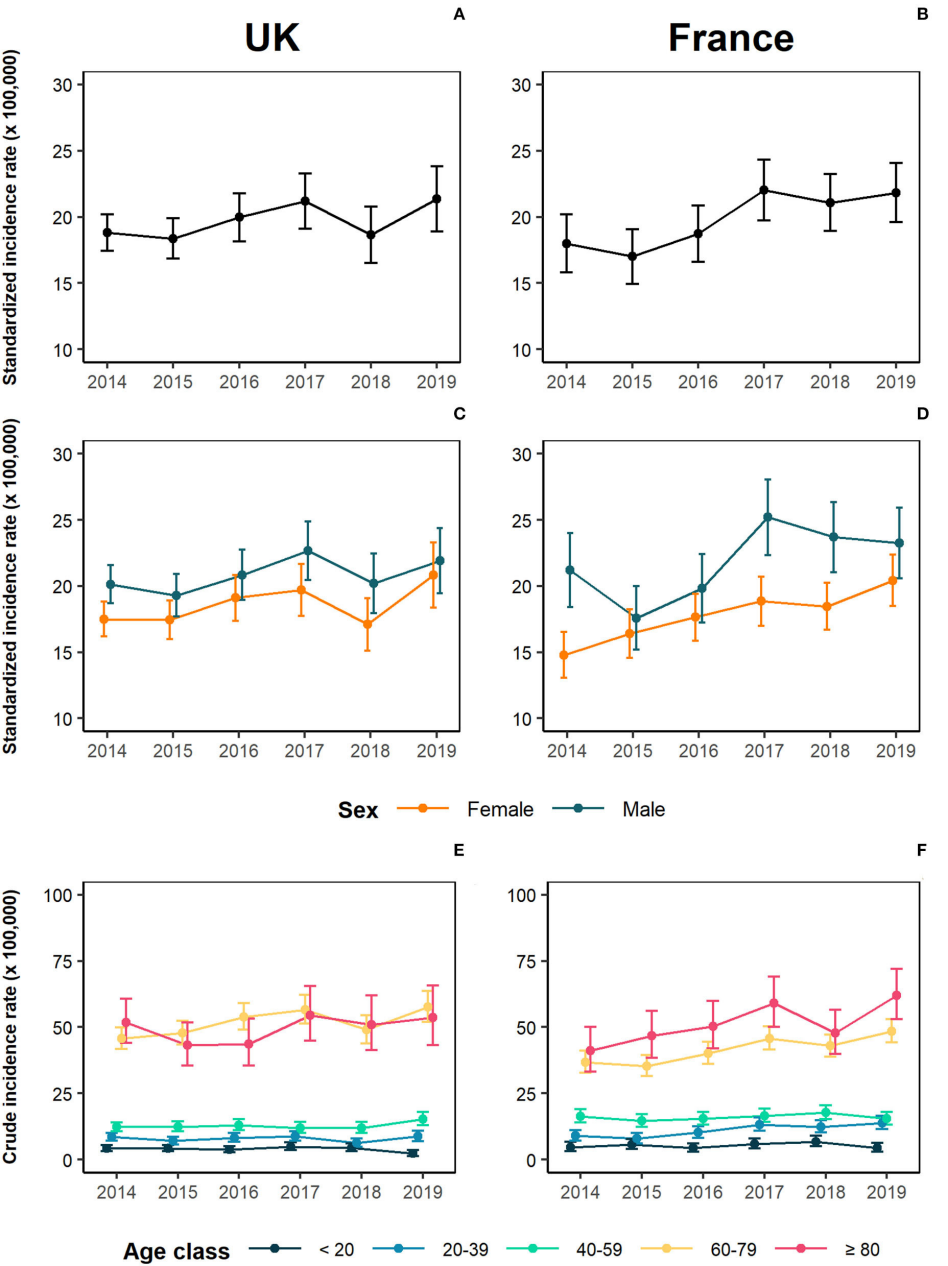
Incidence of essential tremor (ET) incidence in the United Kingdom and France between 2014 and 2019. **(A)** Standardized yearly incidence rates for UK; **(B)** Standardized yearly incidence rates for France; **(C)** Standardized incidence rate for UK, stratified by sex; **(D)** Standardized incidence rates for France, stratified by sex; **(E)** Age-specific rates for UK; **(F)** Age-specific rates for France.

The overall average crude incidence rates (per 100,000 person-years) were 18.20 (95% CI: 15.09–21.32) in the UK and 21.42 (95% CI: 17.83–25.00) in France, whereas the standardized rates were respectively 19.51 (95% CI: 18.97–20.01) and 19.50 (95% CI: 18.97–20.05) (Table 2). From 2014 to 2019, the annual standardized incidence (per 100,000 person-years) slightly increased in both geographical areas, ranging from 18.82 (95% CI: 17.45–20.19) to 21.37 (95% CI: 18.90–23.84) in the UK and from 17.98 (95% CI: 15.8–20.17) to 21.83 (95% CI: 19.59–24.07) in France (Figure 1 and Table 2). This increasing trend remained significant after adjusting for demographic patterns, as confirmed by the results of the regression analyses, which showed a significant and slightly increasing trend both in the UK (IRR for a 1-year variation: 1.03, 95% CI: 1.01–1.04) and in France (IRR for a 1-year variation: 1.06, 95% CI: 1.03–1.08) (Table 3).

**Table 2:** Crude and standardized incidence of essential tremor in the United Kingdom and France between 1 January 2014 and 21 December 2019.

**Year**	**United Kingdom**		**France**	
	**Crude IR** **(x100,000)** **(95% CI)**	**Standardized IR** **(x100,000)** **(95% CI)**	**Crude IR** **(x100,000)** **(95% CI)**	**Standardized IR** **(x100,000)** **(95% CI)**
2014	17.37 (16.31–18.44)	18.82 (17.45–20.19)	18.50 (17.09–19.90)	17.99 (15.80–20.18)
2015	16.97 (15.85–18.08)	18.37 (16.84–19.90)	18.17 (16.80–19.55)	17.00 (14.93–19.08)
2016	18.45 (17.21–19.69)	19.97 (18.157–21.78)	20.31 (18.89–21.74)	18.74 (16.62–20.86)
2017	19.61 (18.26–20.95)	21.19 (19.09–23.28)	23.83 (22.30–25.37)	22.03 (19.74–24.31)
2018	17.38 (16.06–18.69)	18.66 (16.53–20.78)	22.68 (21.20–24.16)	21.08 (18.95–23.22)
2019	20.38 (18.88–21.87)	21.37 (18.91–23.84)	24.61 (23.05–26.16)	21.83 (19.59–24.08)
Yearly average	18.20 (15.09–21.32)	19.514 (18.97–20.06)	21.42 (17.83–25.00)	19.50 (18.95–20.05)

IR, incidence rate; 95%CI, 95% confidence intervals.

**Table 3:** Multivariable Poisson regression analysis for essential tremor incidence in the United Kingdom and France between 2014 and 2019.

	**United Kingdom**		**France**	
	**IRR**	**95% CI**	**IRR**	**95% CI**
**1 year change**	1.03	(1.01–1.04)	1.06	(1.03–1.08)
**Sex**				
Female	Reference	Reference	Reference	Reference
Male	1.12	(1.05–1.19)	1.21	(1.11–1.31)
**Age**				
<20	Reference	Reference	Reference	Reference
20–39	2.00	(1.69–2.39)	2.14	(1.72–2.69)
40–59	3.21	(2.73–3.79)	3.09	(2.51–3.84)
60–79	12.94	(11.13–15.15)	7.98	(6.56–9.81)
≥80	12.61	(10.64–15.00)	9.91	(8.01–12.38)

IRR, incidence rate ratio; 95%CI, 95% confidence intervals.

Results also indicate a higher standardized incidence in males (UK: 20.7 95% CI: 19.9–21.5; France: 21.90, 95% CI: 21.00–22.81) as compared to females (UK: 18.48, 95% CI: 17.7–19.2; France: 17.84, 95% CI: 17.15–18.53) (Figure 1 and Supplementary Table 2). Poisson regression models confirmed a higher incidence rate among males, with an IRR of 1.12 (95%CI: 1.05–1.19) in the UK and of 1.21 (95% CI: 1.11–1.31) in France (Table 3).

The yearly average age-specific incidence rate of ET substantially increased with age, with an abrupt change between the age group 40–59 and the age group 60–79. In the UK, incidence ranged from a minimum of 3.96 (95% CI: 0.95–6.97) in the youngest age group (<20 years) and a maximum of 49.27 (95% CI: 26.29–72.24) in the oldest one (≥80 years), while in France it varied from 5.28 (95% CI: 1.12–9.44) and 51.52 (95% CI: 30.19–72.86) in same age groups (Figure 1 and Supplementary Table 3). The multivariable analysis confirmed the independent effect of age in both countries (Table 3).

Poisson models accounting for the interaction terms highlighted no significant difference in time-trends in sex-specific or age-specific rates, with the exception of the age class 60-79 in the UK, where the increase was more pronounced, with an incidence rate ranging from 45.7 (95% CI: 41.7–49.9) to 57.6 (95% CI: 51.9–63.7) (Figure 1 and Supplementary Tables 3, 4).

The first sensitivity analysis led to the exclusion of 302 cases for the UK and 230 for France. As a consequence, standardized incidence rates were slightly lowered, being respectively 18.34 (95% CI: 17.81–18.87) for UK and 18.68 (95% CI: 18.14–19.22) for France (Supplementary Figure 1 and Supplementary Table 5). When the exclusion period was extended to 3 years, standardized incidence rates for the period 2014–2017 were 18.22 (95% CI: 17.60–18.84) for UK and 17.51 (95% CI: 16.86–18.16) for France (Supplementary Figure 2 and Supplementary Table 6).

## Discussion

This is the largest observational study undertaken so far to estimate ET incidence in Europe, as we based our observation on a population sample of roughly 8.4 million people (UK: 4.6 million, France: 3.8 million). Generally, ET incidence and trends showed similar patterns in UK and France, with a slightly increased incidence of ET throughout the study period. Our findings also reported a higher incidence of ET in males as compared with females in both countries. Age was significantly associated with ET occurrence, with markedly higher rates among individuals aged 60 years or older. Finally, among the selected individuals only a minority had a subsequent diagnosis of Parkinson/parkinsonism within 1 year and 3 years of follow-up that resulted in a slight decrease in ET incidence estimates in both countries after the sensitivity analyses.

To the best of our knowledge, only two studies have investigated the incidence of ET in the general population. The first one was conducted in the US and evaluated the period between 1935 and 1979 in the city of Rochester (61,000 inhabitants), and found high variability of ET incidence across different periods (i.e., 5.8/100,000 inhabitants in the years 1935–1945 vs. 23.7/100,000 inhabitants in the years 1965–1979). The incidence of ET sharply rose with advancing age and reached a peak in individuals aged 80 years or older (84.3/100,000 inhabitants) (12). The second study was the only population-based study conducted in Europe (Spain) to estimate ET incidence: it included 5,022 subjects aged 65 years or more, and it reported an incidence of 616/100,000 person-years in the study cohort with increased incidence by age (11). Previous studies, therefore, indicated higher incidence in the elderly, as compared with our results. However, differences in the data source, study design, and ET definition are likely to explain the observed differences. Indeed, participation in the Spanish study, which focused on three neurological disorders, was voluntary and individuals might have been more prone to participate if they already had some neurological symptoms. Therefore, this selective recruitment might have contributed to overestimating the observed phenomenon (11). The US study, on the other hand, based its observation on administrative records, therefore the data source is more similar to that used for the present study. However, it refers to the past century, and unmeasurable individual factors, as well as physicians' awareness of ET, might have strongly varied during the last 40 years (12). In addition, in the previous studies the impact of potential differences in ET definition, which has changed in the last years, cannot be completely ruled out, and therefore it is reasonable to speculate that some of the ET cases actually were initial signs of other degenerative diseases such as Parkinson's disease (2).

This study benefits from large sample size and the prospective collection of highly standardized healthcare records, that can be considered representative of “real-life” clinical practice. It has been demonstrated that the UK database is representative of the UK population in charge of all general practices in terms of demographic characteristics and prevalence of numerous comorbidities (13). Similarly, patients included in the representative GPs pool of the French database were shown to be representative of the French population in terms of demographic characteristics (21). Therefore, our data are generalizable to the whole UK and French population.

As already highlighted in other studies based on the same database, there are a number of limitations that are intrinsic to the data source. First, the GP database is intended for patient management purposes rather than medical research (22). Therefore, it is possible to speculate that only diagnoses deemed relevant for patients' care were collected. However, it should be recognized that ET is a chronic degenerative disease that requires continuous care, therefore it is implausible that this diagnosis has never been reported in a symptomatic patient's records. Second, in the French cohort, we might not have had access to the full history of some patients if during the study these patients consulted GPs outside the representative panel. Moreover, potential bias in the estimation of incidence could arise from the selection algorithm: although we are confident in the reliability of our results, we could not validate the ET diagnosis reported in the database. Third, we were not able to trace the clinical symptoms of patients before the ET diagnosis (e.g., hand or head tremor) for two reasons: pre-diagnostic features/symptoms are unlikely to be recorded by primary care physicians, and there are no Read or ICD-10 codes specific for the symptoms of interest. This lack of information might have underestimated the true ET incidence if patients with mild symptoms wait years before seeking medical care. Moreover, misclassification of ET diagnosis might also occur if Parkinson's disease/parkinsonism was not considered by clinicians during the study period. Finally, the use of different coding vocabularies in the UK (Read Code) and France (ICD-10) might have led to a differential selection in the two countries. However, the strong similarities in the overall and the stratified estimates, as well as in the temporal trends, support the validity and generalizability of our results.

In conclusion, this study provides the most recent estimates of ET incidence in two large samples of the European population and outlines the increasing trend during the observed years, particularly among elder patients. As the world's population ages, with individuals aged 60 years or older projected to reach 2.1 billion by 2050 (23), the burden of this condition will certainly rise. Considering that individuals with ET experience functional disability, diminished quality of life, and in some case have a cognitive impairment, depression, or other comorbidities (24–27), further studies should be conducted to inform policy-makers on the burden of this disease not only in terms of clinical characteristics of ET but also in terms of healthcare resources utilization and associated costs of these patients.

## Data availability statement

The data used in the preparation of this article are available from the Cegedim company upon 253 reasonable request (info@the-health-improvement-network.co.uk).

## Ethics statement

The study was approved by the THIN Scientific Research Committee (SRC) on 6th July 2021 (SRC reference 21-014). Written informed consent from the patients/participants or patients/participants' legal guardian/next of kin was not required to participate in this study in accordance with the national legislation and the institutional requirements.

## Author contributions

IA, SC, and GM study concept and design, analysis, interpretation of data, drafting of the manuscript, and statistical analysis. SC and DR statistical analysis. CE-P, CP, LG, and DV data extraction. LM and CF critical revision of the manuscript for important intellectual content. All authors contributed to the article and approved the submitted version.

## Conflict of interest

Author LM has received grants and personal fees from Bayer AG, Boehringer Ingelheim, Pfizer and Daiichi-Sankyo. Authors CE-P, CP, LG, and DV are employed by Cegedim. The remaining authors declare that the research was conducted in the absence of any commercial or financial relationships that could be construed as a potential conflict of interest.

## Publisher's note

All claims expressed in this article are solely those of the authors and do not necessarily represent those of their affiliated organizations, or those of the publisher, the editors and the reviewers. Any product that may be evaluated in this article, or claim that may be made by its manufacturer, is not guaranteed or endorsed by the publisher.
