# Supplementary material for: Time trends in the incidence of essential tremor: Evidences from UK and France primary care data
Source: Front Neurol. 2022 Sep 20;13:987618. doi: 10.3389/fneur.2022.987618 (PMC9531026; doi:10.3389/fneur.2022.987618)
Supplement: Supplementary file 1 [file Data_Sheet_1.docx]

Supplementary Material

**Supplementary Table 1a.** List of read codes used to identify dystonia, ataxia, Parkinson’s disease and parkinsonisms in UK database.

| **Read code** | **Read term** |
| --- | --- |
| **Dystonia** | |
| 7Q04000 | Torsion dystonias other involuntary movements drugs Band 1 |
| F136.00 | Idiopathic torsion dystonia |
| F136000 | Idiopathic familial dystonia |
| F137.00 | Symptomatic torsion dystonia |
| F137200 | Drug-induced dystonia |
| F137y00 | Other specified symptomatic torsion dystonia |
| F137z00 | Symptomatic torsion dystonia NOS |
| F138.00 | Fragments of torsion dystonia |
| F138z00 | Fragments of torsion dystonia NOS |
| F13A.00 | Paroxysmal dystonia |
| F13B.00 | Myoclonic dystonia |
| F13X.00 | Dystonia, unspecified |
| Fyu2400 | [X]Other dystonia |
| Fyu2A00 | [X]Dystonia, unspecified |
| **Ataxia** | |
| 2993.00 | O/E - gait ataxic |
| 29L2.00 | O/E - arms ataxic |
| 29L3.00 | O/E - legs ataxic |
| F11y100 | Cerebral ataxia |
| F140.00 | Friedreich's ataxia |
| F142000 | Marie's cerebellar ataxia |
| F143.00 | Cerebellar ataxia NOS |
| F144.00 | Cerebellar ataxia in diseases EC |
| F144000 | Cerebellar ataxia due to alcoholism |
| F144200 | Cerebellar ataxia due to neoplasia |
| F144z00 | Cerebellar ataxia in disease NOS |
| F145.00 | Congenital nonprogressive ataxia |
| F146.00 | Early onset cerebellar ataxia with hypogonadism |
| F14y000 | Ataxia-telangiectasia |
| F1y0.00 | Fragile X associated tremor ataxia syndrome |
| F23y000 | Ataxic infantile cerebral palsy |
| F23y400 | Ataxic diplegic cerebral palsy |
| F365.00 | Neuropathy in association with hereditary ataxia |
| Fyu1000 | [X]Other hereditary ataxias |
| G558000 | Cardiomyopathy in Friedreich's ataxia |
| R012000 | [D]Ataxic gait |
| R013000 | [D]Ataxia NOS |
| **Parkinson’s disease and parkinsonisms** | |
| 2987.00 | O/E -Parkinson flexion posture |
| 2994.00 | O/E-festination-Parkinson gait |
| 073D.00 | Parking/toll fee collector |
| 13Y5.00 | Parkinsons Society member |
| 147F.00 | History of Parkinson's disease |
| 1I8..00 | Parkinson's disease excluded |
| 297A.00 | O/E - Parkinsonian tremor |
| 38GM.00 | Lindop Parkinson's assessment scale |
| 43rP.00 | Anti basal ganglia antibody level |
| 8T06.00 | Referral to Parkinson's service |
| A94y100 | Syphilitic parkinsonism |
| B510000 | Malignant neoplasm of basal ganglia |
| Eu02300 | [X]Dementia in Parkinson's disease |
| F11x900 | Cerebral degeneration in Parkinson's disease |
| F12..00 | Parkinson's disease |
| F120.00 | Paralysis agitans |
| F121.00 | Parkinsonism secondary to drugs |
| F123.00 | Postencephalitic parkinsonism |
| F124.00 | Vascular parkinsonism |
| F12W.00 | Secondary parkinsonism due to other external agents |
| F12X.00 | Secondary parkinsonism, unspecified |
| F12z.00 | Parkinson's disease NOS |
| F130.00 | Other basal ganglia degenerative diseases |
| F130300 | Parkinsonism with orthostatic hypotension |
| F130z00 | Other basal ganglia degenerative disease NOS |
| Fyu2000 | [X]Other drug-induced secondary parkinsonism |
| Fyu2100 | [X]Other secondary parkinsonism |
| Fyu2200 | [X]Parkinsonism in diseases classified elsewhere |
| Fyu2300 | [X]Other specified degenerative diseases/the basal ganglia |
| Fyu2900 | [X]Secondary parkinsonism, unspecified |
| Fyu2B00 | [X]Secondary parkinsonism due to other external agents |
| G567400 | Wolff-Parkinson-White syndrome |
| G64z400 | Infarction of basal ganglia |
| SL6..00 | Anticonvulsant and antiParkinsonian drug poisoning |
| SL6y.00 | Antiparkinsonism drug poisoning |
| SL6yz00 | Antiparkinsonian drug poisoning NOS |
| SL6z.00 | Anticonvulsant or antiparkinsonian drug poisoning NOS |
| T7yB.00 | Place of occurrence of accident or poisoning, parking lot |
| T7yC.00 | Place of occurrence of accident or poisoning, parking place |
| T850.00 | Accidental poisoning by anticonvulsant + anti-parkinson drug |
| T850z00 | Accidental poisoning by anticonvulsant/anti-parkin drug NOS |
| TJ6..00 | Adverse reaction to anticonvulsants/anti-parkinsonism drugs |
| TJ64.00 | Adverse reaction to antiparkinsonism drugs |
| TJ64z00 | Adverse reaction to antiparkinsonism drugs NOS |
| TJ6z.00 | Adverse reaction to anticonvulsant/antiparkinsonism drug NOS |
| U1A3.00 | [X]Accident poisoning/exposure to antiparkinson drug |
| U1A3500 | [X]Accid poison/expos antiparkinson drug trade/service area |
| U606700 | [X]Antiparkinsonism drugs caus advers effects in therap use |

**Supplementary Table 1b.** List of read codes used to identify dystonia, ataxia, Parkinson’s disease and parkinsonisms in France database.

| **ICD-10-CM code** | **ICD-10-CM term** |
| --- | --- |
| **Dystonia** | |
| G24.3 | Spasmodic torticollis |
| G24.0 | Drug induced dystonia |
| G24.1 | Genetic torsion dystonia |
| G24.2 | Idiopathic nonfamilial dystonia |
| G24.8 | Other dystonia |
| G24.9 | Dystonia, unspecified |
| **Ataxia** | |
| F44.4 | Conversion disorder with motor symptom or deficit |
| G11 | Hereditary ataxia |
| G11.1 | Early-onset cerebellar ataxia |
| G11.10 | Early-onset cerebellar ataxia, unspecified |
| G11.11 | Friedreich ataxia |
| G11.19 | Other early-onset cerebellar ataxia |
| G11.2 | Late-onset cerebellar ataxia |
| G11.3 | Cerebellar ataxia with defective DNA repair |
| G11.4 | Hereditary spastic paraplegia |
| G11.8 | Other hereditary ataxias |
| G11.9 | Hereditary ataxia, unspecified |
| G32.81 | Cerebellar ataxia in diseases classified elsewhere |
| G60.2 | Neuropathy in association with hereditary ataxia |
| I69.093 | Ataxia following nontraumatic subarachnoid hemorrhage |
| I69.193 | Ataxia following nontraumatic intracerebral hemorrhage |
| I69.293 | Ataxia following other nontraumatic intracranial hemorrhage |
| I69.393 | Ataxia following cerebral infarction |
| I69.893 | Ataxia following other cerebrovascular disease |
| I69.993 | Ataxia following unspecified cerebrovascular disease |
| R26.0 | Ataxic gait |
| R27.0 | Ataxia, unspecified |
| R27.8 | Other lack of coordination |
| R27.9 | Unspecified lack of coordination |
| **Parkinson’s disease and parkinsonisms** | |
| G20 | Parkinson's disease |
| G21 | Secondary parkinsonism, unspecified |
| G21.1 | Other drug-induced secondary parkinsonism |
| G21.11 | Neuroleptic induced parkinsonism |
| G21.19 | Other drug induced secondary parkinsonism |
| G21.2 | Secondary parkinsonism due to other external agents |
| G21.3 | Postencephalitic parkinsonism |
| G21.4 | Vascular parkinsonism |
| G21.8 | Other secondary parkinsonism |
| G90.3 | Multi-system degeneration of the autonomic nervous system |

**Supplementary Table 2.** Standardized incidence of Essential Tremor, by sex, in United Kingdom and France between January 1^st^, 2014 and December 21^st^, 2019

|  | **Crude incidence rate (x100,000)**  **(95% Confidence Interval)** | | **Standardized incidence rate (x100,000)**  **(95% Confidence Interval)** | |
| --- | --- | --- | --- | --- |
|  | **Female** | **Male** | **Female** | **Male** |
| **United Kingdom** | | | | |
| **2014** | 16.78 (15.31-18.24) | 17.99 (16.45-19.52) | 17.50 (16.20-18.81) | 20.14 (18.70-21.57) |
| **2015** | 16.65 (15.10-18.20) | 17.29 (15.69-18.89) | 17.45 (15.99-18.90) | 19.29 (17.68-20.89) |
| **2016** | 18.12 (16.39-19.85) | 18.79 (17.01-20.57) | 19.10 (17.37-20.82) | 20.85 (18.95-22.74) |
| **2017** | 18.96 (17.10-20.81) | 20.27 (18.33-22.20) | 19.70 (17.74-21.67) | 22.67 (20.45-24.89) |
| **2018** | 16.46 (14.66-18.26) | 18.31 (16.39-20.22) | 17.10 (15.10-19.09) | 20.22 (17.96-22.47) |
| **2019** | 20.33 (18.22-22.44) | 20.43 (18.29-22.56) | 20.84 (18.38-23.29) | 21.91 (19.44-24.39) |
| **Yearly average** | 17.72 (13.40-22.05) | 18.70 (14.22-23.17) | 18.48 (17.74-19.21) | 20.70 (19.89-21.52) |
| **France** | | | | |
| **2014** | 15.76 (14.06-17.47) | 22.20 (19.84-24.55) | 14.79 (13.07-16.52) | 21.19 (18.39-23.98) |
| **2015** | 17.89 (16.09-19.69) | 18.547 (16.42-20.68) | 16.42 (14.58-18.26) | 17.59 (15.20-19.98) |
| **2016** | 19.48 (17.64-21.32) | 21.45 (19.20-23.70) | 17.64 (15.86-19.41) | 19.83 (17.25-22.42) |
| **2017** | 21.27 (19.36-23.17) | 27.36 (24.83-29.89) | 18.86 (16.99-20.72) | 25.20 (22.34-28.06) |
| **2018** | 20.68 (18.82-22.54) | 25.41 (22.99-27.83) | 18.46 (16.71-20.22) | 23.70 (21.05-26.35) |
| **2019** | 23.72 (21.70-25.73) | 25.81 (23.36-28.26) | 20.43 (18.49-22.37) | 23.24 (20.58-25.90) |
| **Yearly average** | 19.87 (15.32-24.42) | 23.52 (17.74-29.30) | 17.84 (17.15-18.53) | 21.90 (20.98-22.81) |

**Supplementary Table 3.** Crude incidence Rate of Essential Tremor, by age, in United Kingdom and France between January 1^st^, 2014 and December 21^st^, 2019

|  | **United Kingdom** | | | | | **France** | | | | |
| --- | --- | --- | --- | --- | --- | --- | --- | --- | --- | --- |
|  | **Crude incidence rate (x100,000)** | | | | | **Crude incidence rate (x100,000)** | | | | |
|  | *Age class* | | | | | *Age class* | | | | |
|  | **<20** | **20-39** | **40-59** | **60-79** | **≥80** | **<20** | **20-39** | **40-59** | **60-79** | **≥80** |
| **2014** | 4.12  (3.09-5.37) | 8.46  (7.10-10) | 12.25  (10.62-14.07) | 45.65  (41.67-49.91) | 51.83  (43.99-60.66) | 4.66  (3.19-6.58) | 8.88  (7.02-11.08) | 16.33  (13.92-19.03) | 36.75  (32.71-41.16) | 41.06  (33.22-50.19) |
| **2015** | 4.13  (3.05-5.48) | 7.05  (5.74-8.56) | 12.4  (10.65-14.35) | 47.70  (43.39-52.33) | 43.12  (35.53-51.84) | 5.64  (4.01-7.71) | 7.88  (6.14-9.95) | 14.66  (12.41-17.20) | 35.3  (31.42-39.53) | 46.72  (38.47-56.21) |
| **2016** | 3.72  (2.63-5.10) | 8.2  (6.70-9.93) | 13.02  (11.11-15.18) | 53.85  (48.95-59.09) | 43.66  (35.49-53.16) | 4.29 (  2.89-6.12) | 10.15  (8.19-12.44) | 15.50  (13.22-18.05) | 40.09  (36.08-44.43) | 50.36  (42.04-59.84) |
| **2017** | 4.75  (3.45-6.37) | 8.80  (7.18-10.68) | 11.95  (10.02-14.13) | 56.62  (51.37-62.26) | 54.53  (44.90-65.62) | 5.92  (4.25-8.03) | 13.19  (10.95-15.76) | 16.57  (14.22-19.19) | 45.77  (41.54-50.3) | 59.00  (50.13-68.99) |
| **2018** | 4.46  (3.16-6.12) | 6.25  (4.85-7.94) | 11.92  (9.92-14.20) | 48.97  (43.93-54.41) | 50.83  (41.22-62.01) | 6.75  (4.99-8.92) | 12.34  (10.17-14.83) | 17.72  (15.30-20.42) | 42.91  (38.88-47.25) | 47.69  (39.88-56.59) |
| **2019** | 2.23  (1.30-3.58) | 8.75  (6.98-10.83) | 15.27  (12.88-17.98) | 57.57  (51.88-63.72) | 53.56  (43.18-65.69) | 4.37  (2.97-6.20) | 13.72  (11.39-16.4) | 15.45  (13.17-18.01) | 48.44  (44.15-53.02) | 61.98  (53.11-71.91) |
| **Average** | 3.96  (0.95 - 6.97) | 7.92  (4.04-11.80) | 12.70  (7.72-17.68) | 51.13  (38.85-63.42) | 49.27  (26.29-72.24) | 5.28  (1.12-9.44) | 11.04  (5.73-16.35) | 16.05  (10.12-21.98) | 41.81  (31.55-52.06) | 51.52  (30.19-72.86) |

**Supplementary Table 4.** Results of the Poisson regression models accounting for the interactions between sex and calendar year and age and calendar year.

|  | **United Kingdom** | | | | **France** | | | |
| --- | --- | --- | --- | --- | --- | --- | --- | --- |
|  | ***Beta*** | ***Standard Error*** | ***Z value*** | ***P-value*** | ***Beta*** | ***Standard Error*** | ***Z value*** | ***P-value*** |
| Intercept | 72.18 | 79.72 | 0.91 | 0.37 | -66.58 | 118.50 | -0.56 | 0.58 |
| Year | -0.04 | 0.04 | -1.03 | 0.30 | 0.03 | 0.06 | 0.48 | 0.64 |
| Sex (Male) | 10.27 | 33.51 | 0.31 | 0.76 | 33.17 | 49.80 | 0.67 | 0.51 |
| Age 20-39 | -79.07 | 91.64 | -0.86 | 0.39 | -170.60 | 135.90 | -1.26 | 0.22 |
| Age 40-59 | -135.60 | 86.60 | -1.57 | 0.12 | 20.61 | 127.60 | 0.16 | 0.87 |
| Age 60-79 | -162.50 | 81.10 | -2.00 | 0.05 | -78.26 | 121.10 | -0.65 | 0.52 |
| Age ≥ 80 | -124.60 | 90.15 | -1.38 | 0.17 | -84.60 | 130.90 | -0.65 | 0.52 |
| Year * Sex (Male) | -0.01 | 0.02 | -0.30 | 0.76 | -0.02 | 0.02 | -0.66 | 0.51 |
| Year * Age 20-39 | 0.04 | 0.05 | 0.87 | 0.38 | 0.08 | 0.07 | 1.26 | 0.21 |
| Year * Age 40-59 | 0.07 | 0.04 | 1.58 | 0.11 | -0.01 | 0.06 | -0.15 | 0.88 |
| Year * Age 60-79 | 0.08 | 0.04 | 2.04 | 0.04 | 0.04 | 0.06 | 0.66 | 0.51 |
| Year * Age ≥ 80 | 0.06 | 0.04 | 1.41 | 0.16 | 0.04 | 0.06 | 0.66 | 0.51 |

**Supplementary Figure 1.** Incidence of essential tremor (ET) incidence in United Kingdom and France between 2014-2019. The analysis includes only those without Parkinson’s disease and parkinsonisms within 1 year of follow-up.

**
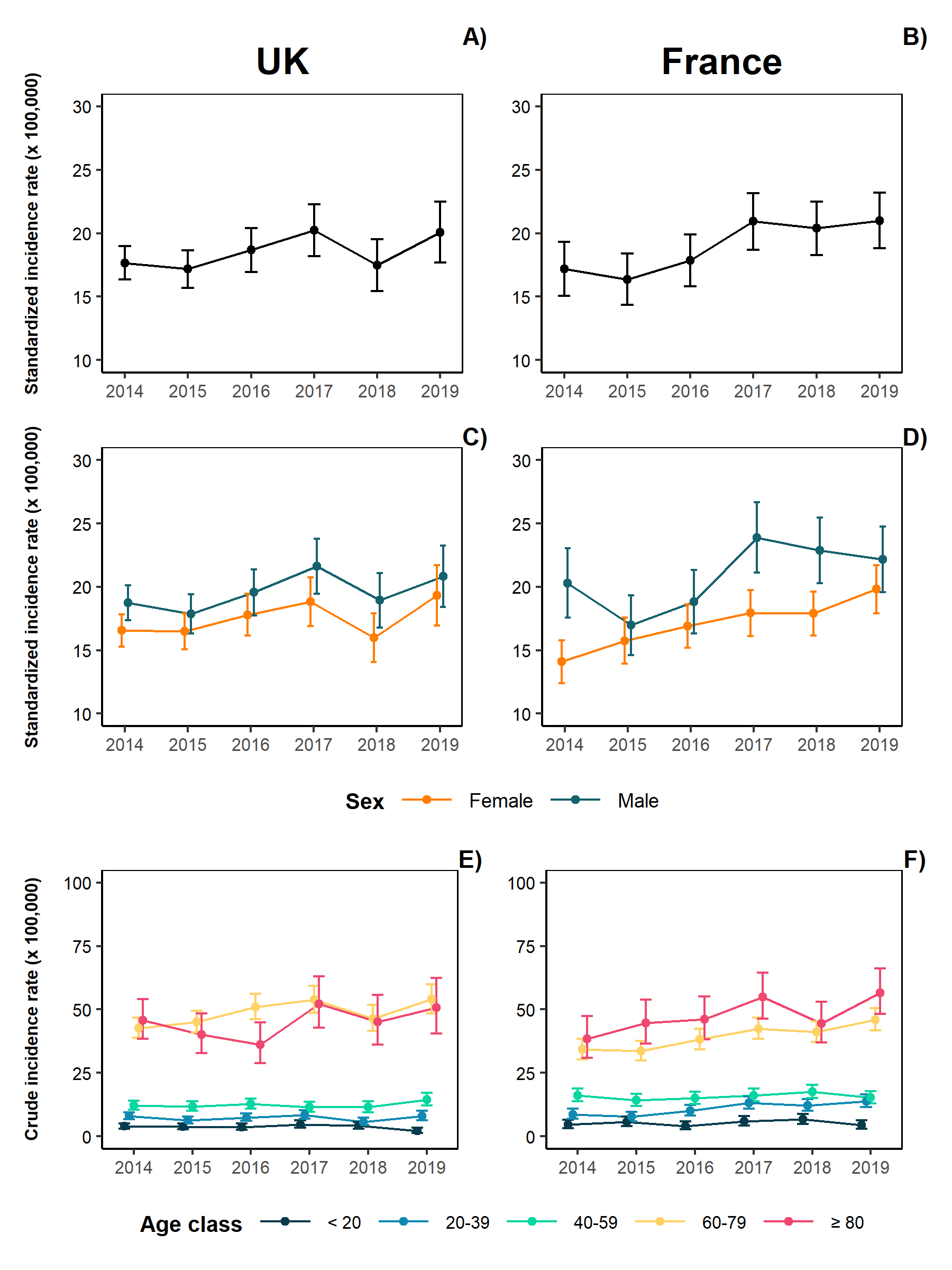
**

**Supplementary Table 5.** Crude and Standardized incidence of Essential Tremor, in United Kingdom and France between January 1^st^, 2014 and December 21^st^, 2019. The analysis includes only those without Parkinson’s disease and parkinsonisms within 1 year of follow-up.

| **Year** | **United Kingdom** | | **France** | |
| --- | --- | --- | --- | --- |
|  | **Crude IR**  **(x100,000)**  **(95% CI)** | **Standardized IR**  **(x100,000)**  **(95% CI)** | **Crude IR**  **(x100,000)**  **(95% CI)** | **Standardized IR**  **(x100,000)**  **(95% CI)** |
| **2014** | 16.30 (15.27-17.32) | 17.65 (16.34-18.97) | 17.61 (16.24-18.98) | 17.19 (15.05-19.33) |
| **2015** | 15.86 (14.78-16.94) | 17.17 (15.70-18.65) | 17.44 (16.09-18.79) | 16.36 (14.33-18.39) |
| **2016** | 17.23 (16.03-18.43) | 18.68 (16.94-20.41) | 19.35 (17.96-20.74) | 17.86 (15.81-19.91) |
| **2017** | 18.72 (17.41-20.03) | 20.23 (18.18-22.28) | 22.57 (21.08-24.07) | 20.92 (18.70-23.14) |
| **2018** | 16.27 (14.99-17.54) | 17.46 (15.42-19.50) | 21.87 (20.41-23.33) | 20.38 (18.29-22.47) |
| **2019** | 19.12 (17.66-20.57) | 20.07 (17.68-22.47) | 23.55 (22.03-25.08) | 20.99 (18.81-23.18) |
| **Yearly average** | 17.10 (14.08-20.11) | 18.34 (17.81-18.87) | 20.47 (16.96-23.97) | 18.68 (18.14-19.22) |

IR: Incidence Rate; 95%CI: 95% confidence intervals

**Supplementary Figure 2.** Incidence of essential tremor (ET) incidence in United Kingdom and France between 2014-2017. The analysis includes only those without Parkinson’s disease and parkinsonisms within 3 years of follow-up.

**
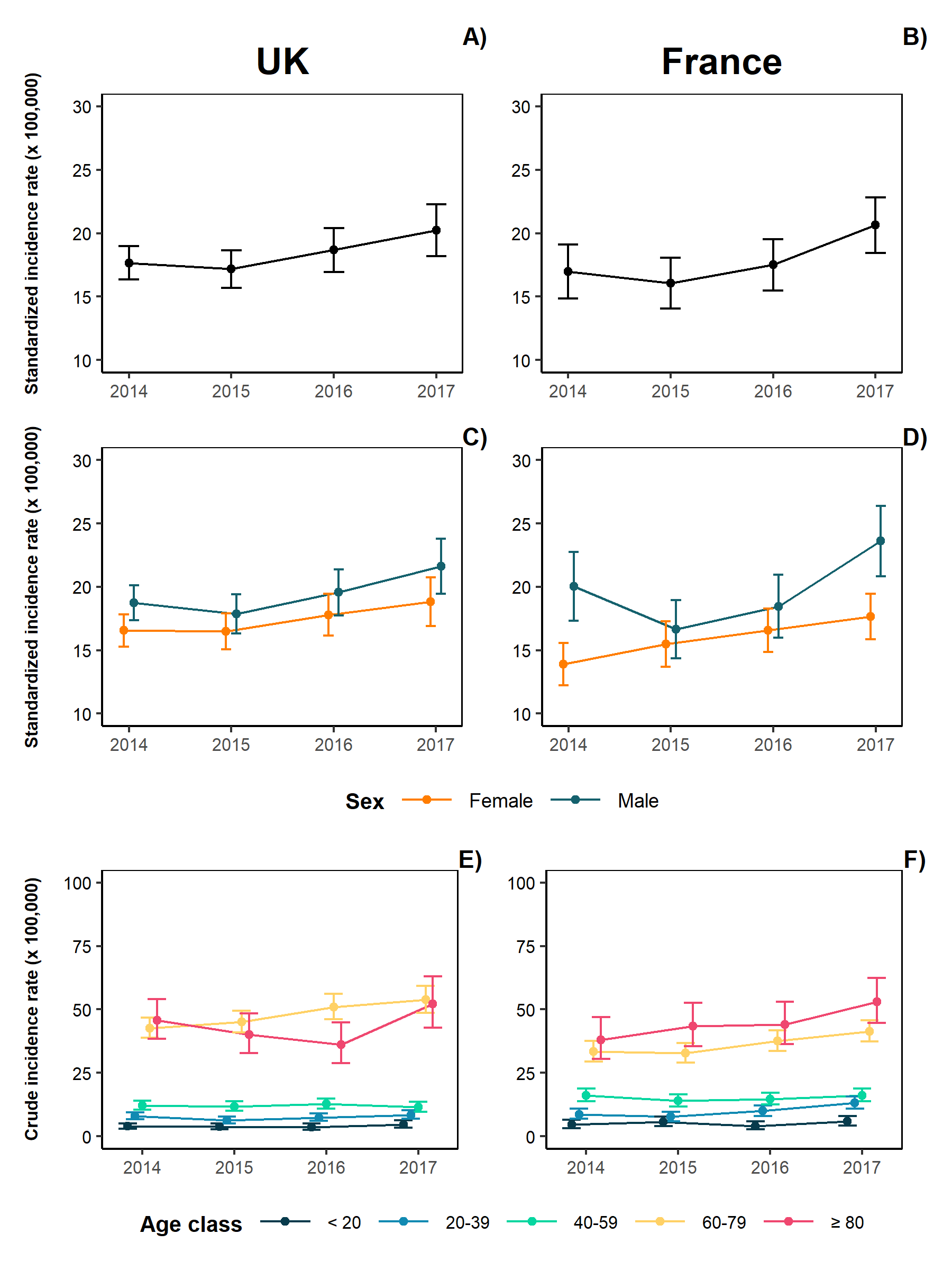
**

**Supplementary Table 6.** Crude and Standardized incidence of Essential Tremor, in United Kingdom and France between January 1^st^, 2014 and December 21^st^, 2017. The analysis includes only those without Parkinson’s disease and parkinsonisms within 3 years of follow-up.

| **Year** | **United Kingdom** | | **France** | |
| --- | --- | --- | --- | --- |
|  | **Crude IR**  **(x100,000)**  **(95% CI)** | **Standardized IR**  **(x100,000)**  **(95% CI)** | **Crude IR**  **(x100,000)**  **(95% CI)** | **Standardized IR**  **(x100,000)**  **(95% CI)** |
| **2014** | 16.30 (15.27-17.32) | 17.65 (16.34-18.97) | 17.39 (16.03-18.75) | 16.98 (14.86-19.11) |
| **2015** | 15.86 (14.78-16.94) | 17.17 (15.70-18.65) | 17.11 (15.78-18.45) | 16.07 (14.06-18.08) |
| **2016** | 17.23 (16.03-18.43) | 18.68 (16.94-20.41) | 18.96 (17.58-20.34) | 17.51 (15.48-19.54) |
| **2017** | 18.72 (17.41-20.03) | 20.23 (18.18-22.28) | 22.21 (20.73-23.69) | 20.64 (18.43-22.84) |
| **Yearly average** | 16.91 (14.59-19.22) | 18.22 (17.60-18.84) | 18.97 (16.19-21.75) | 17.51 (16.86-18.16) |

IR: Incidence Rate; 95%CI: 95% confidence intervals
